# Transcriptome analysis revealed the roles of long non-coding RNA and mRNA in the bursa of Fabricius during pigeon (*Columba livia*) development

**DOI:** 10.3389/fimmu.2022.916086

**Published:** 2022-07-25

**Authors:** Xun Wang, Jie Wu, Silu Hu, Qiyi Peng, Fuxing Yang, Ling Zhao, Yu Lin, Qianzi Tang, Long Jin, Jideng Ma, Hongrui Guo, Huaqiao Tang, Anan Jiang, Xuewei Li, Mingzhou Li

**Affiliations:** ^1^ Institute of Animal Genetics and Breeding, College of Animal Science and Technology, Sichuan Agricultural University, Chengdu, China; ^2^ Farm Animal Genetic Resources Exploration and Innovation Key Laboratory of Sichuan Province, Sichuan Agricultural University, Chengdu, China; ^3^ College of Veterinary Medicine, Sichuan Agricultural University, Chengdu, China

**Keywords:** pigeon, bursa of Fabricius, RNA-seq, lncRNA, mRNA, development

## Abstract

The bursa of Fabricius (BF) is the critical humoral immune organ to birds, playing an essential role in B lymphocyte differentiation. However, unlike other poultries, surgical removal of pigeon BF did not limit humoral immune responsiveness. To investigate the expression profiles and the potential role of mRNA and long non-coding RNA (LncRNA) in squab BFs, transcriptome analysis was performed by RNA-Sequencing (RNA-Seq) over three developmental stages (1-day, 13 and 26 days old). We identified 13,072 mRNAs and 19,129 lncRNAs, of which 2,752 mRNAs and 1,515 lncRNAs were differential expressed (DE) in pigeon BFs over three developmental stages. Cluster analysis presented different expression patterns in DE mRNAs and lncRNAs. Functional enrichment analysis revealed that DE lncRNAs and mRNAs with distinct expression patterns might play crucial roles in the immune system process and tissue morphogenesis. In particular, some DE genes and lncRNAs with higher expression levels in 13D or 26D are related to lymphocyte activation and differentiation, adaptive immune response, positive regulation of immune response, leukocyte migration, etc. Protein-protein interaction (PPI) network and Molecular Complex Detection (MCODE) analysis sreened six significant modules containing 37 genes from immune-related DE gene cluster, which is closely linked in B cell activation, lymphocyte differentiation, B cell receptor signaling pathway, etc. Our study characterizes mRNA and lncRNA transcriptomic variability in pigeon BFs over different developmental stages and enhances understanding of the mechanisms underlying physiological functions of pigeon BF.

## Introduction

Mammals and birds evolved from a common reptilian ancestor and have many common immunological systems (1). Meanwhile, they also developed some distinctly different characteristics. For instance, unlike human beings and mice, bird B-cells develop in the bursa of Fabricius (2). The bursa of Fabricius, an obscure sac-like structure attached to the proctodeal region of the cloaca (1), is the central humoral immune organ unique to birds (3). BF is responsible for the amplification and differentiation of B lymphoid progenitors within its follicular microenvironment (4). Fully differentiated B lymphocytes migrate from BF to peripheral lymphoid organs to colonize, reproduce and perform important immune functions (5). Of note, BF contains several biologically-active factors, including bursin, brusal peptide 11 (BP11), bursal pentapeptide I (BPP-I), etc. These factors are involved in B cell differentiation, regulation of B-cell development, antigen-specific immune responses (6), antiproliferation of tumor cell, and immunomodulatory activity (7). Due to the importance of BF in B-cell development and immunoglobulin production, surgical removal or pathological changes in BF would result in reduction of humoral immunocompetence (8), and an increase in the susceptibility to infections at certain birds (9). Additionally, BF size and weight might influence the immunity of avian (10, 11).

In China, the pigeon (*Columba livia*) is an indispensable agricultural economic animal for meat and eggs, and has become the fourth-largest domestic poultry by breeding scale, following chicken, duck and goose. Pigeon squabs are altrices, and have an extraordinarily high growth rate. Pigeon BF during development undergoes hypertrophia and hyperplasia of lobules constituting the plicae, and the gradual and continuous increasing of lymphocytes (12). After treatment with cyclophosphamide, pigeon BF produced a rapid atrophy and an intense depletion of lymphoid cells (13). However, unlike other poultries (chickens, White Pekin ducks, Japanese quail, etc.), bursectomy of pigeon in early neonatal period did not affect the development of humoral immune responsiveness (14). This phenomenon indicates that the pigeon BF development process may be different from other poultries in some way.

Chicken BF transcriptome research across distinct developmental stages indicated that DE genes are related in defence response and some signal pathways (Jak-STAT, TLR, Wnt and MAPK, etc) might be involving in the regulation of BF development process (2). Additionally, long non-coding RNAs (lncRNAs) have been found to play crucial roles during organ development in recent years (15). lncRNAs are defined as non-protein coding transcripts longer than 200 nucleotides (16), which regulate gene expression at the epigenetic, transcriptional and post-transcriptional levels *via* different mechanisms (17). To date, lncRNAs and mRNAs closely linked to the functional development of pigeon BF have not been identified. Here, we aim to explore the lncRNA and mRNA expression profiles in BF and their potential roles during pigeon development by RNA sequencing technology. Our findings will provide insights into the transcriptional variations in pigeon BF development and enhance the understanding physiological functions of squab BF.

## Results

### Phenotypic measurements

**Figure 1:**
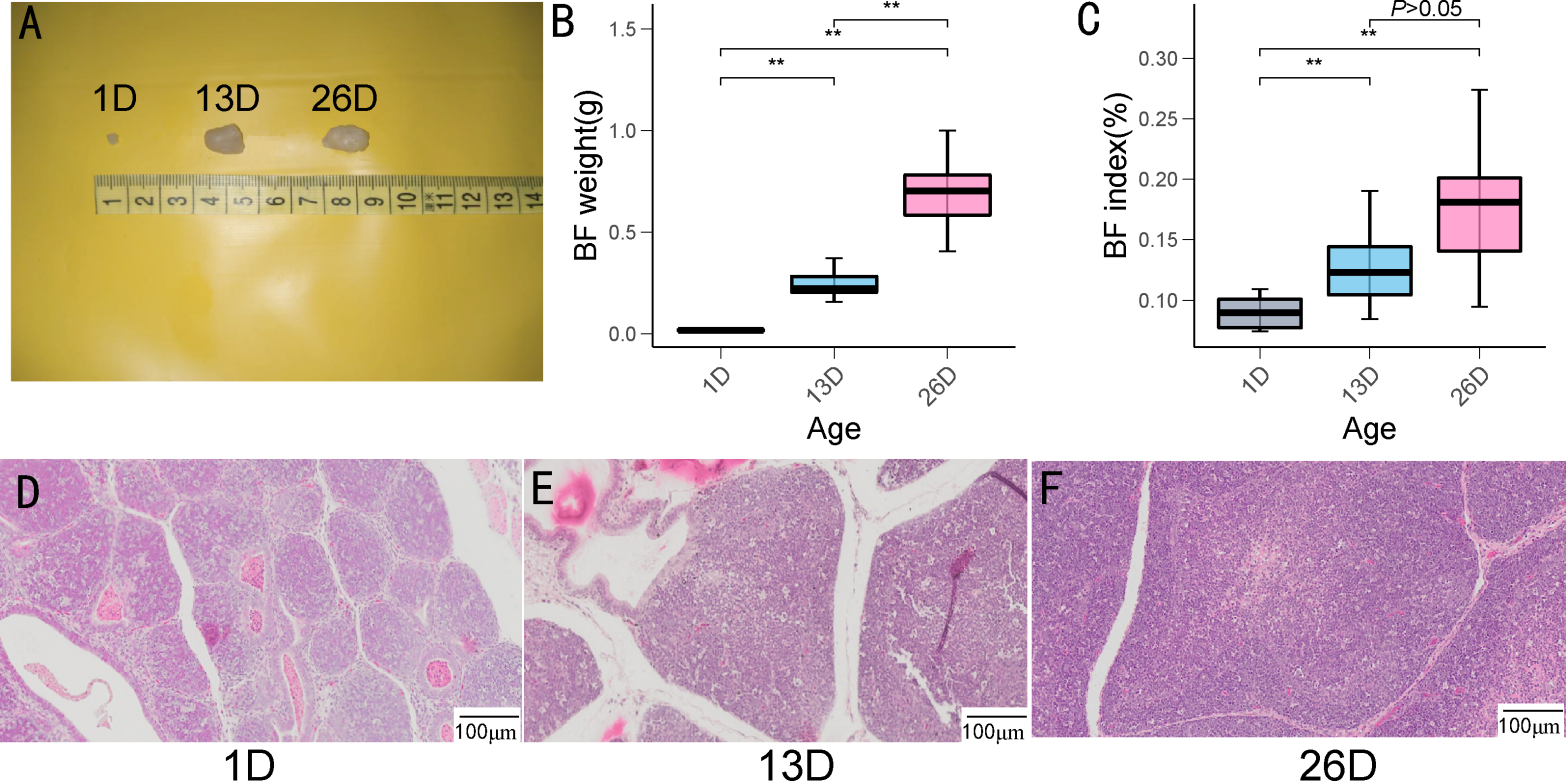
BF weight, BF index and histomorphological changes at the different development stages of pigeon squabs. **(A)**.BF size **(B)**. BF weight **(C)**. BF index (n = 13) **(D-F)**. Histomorphological characteristics in pigeon BF over the different development stages. ** indicates P < 0.01.

In this study, we investigated morphological changes, BF weight, and BF index across three different development stages of pigeons. As depicted in **Figure 1A– C**, BF size and weight conspicuously increased from 1-day-old to 26-day-old. BF indexes in 13-day-old and 26-day-old squabs are conspicuously higher than those of 1-day-old pigeons. However, BF indexes did not exhibit a significant difference between 13-day-old and 26-day-old pigeon squabs. From the results of BF morphological changes, lymphoid follicles in BFs gradually enlarged from 1-day-old to 26-day-old (**Figure 1D– F**).

### Transcriptome sequencing and assembly

To systematically investigate lncRNA and mRNA expression in BF during pigeon development, we yielded a total of 103.56 Gb raw sequence data from 9 libraries (each stage constructed 3 libraries). After quality control, 340.91 million clean reads (102.27 Gb data) were retained for further analysis. Approximately 81.51~94.02% of the clean data were mapped to the pigeon genome (**Supplementary Table 2**).

**Figure 2:**
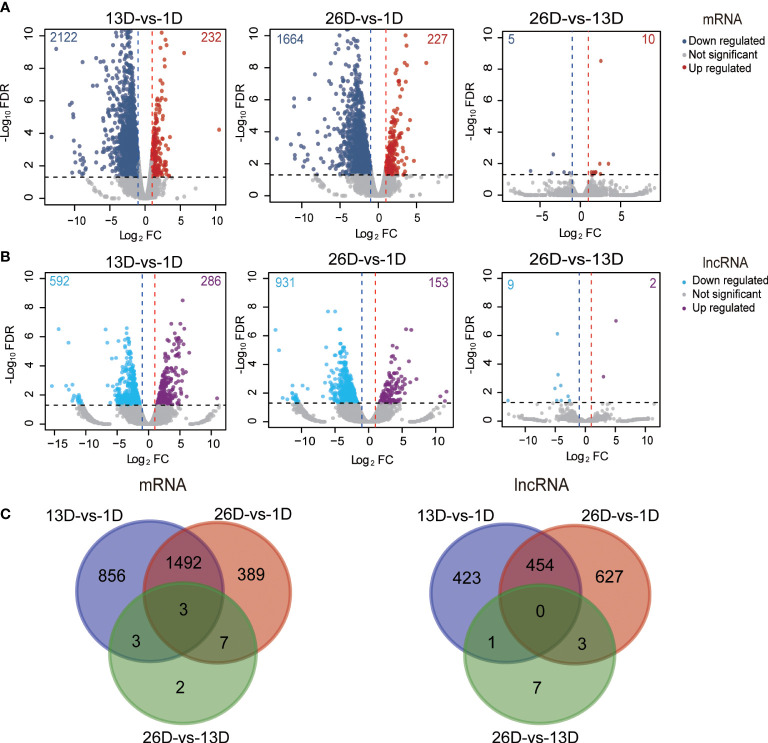
Differentially expressed mRNAs and lncRNAs during squab BF development. **(A–B)**. Volcano plot of the DE mRNAs(A) and lncRNAs **(B)** between two different development stages. The x-axis indicates the difference in expression level on a log2 (fold change). The y-axis represents the corresponding false discovery rate on a negative log 10(FDR). **(C)**. Venn plot shows the number of overlapping DE mRNAs and lncRNAs in different developmental stages.

In total, 13,072 mRNAs and 19,129 lncRNAs (containing 2,540 known and 16,589 novel lncRNAs) were identified in pigeon BFs during development (**Supplementary Table 3**). Based on characteristics comparisons with mRNAs, lncRNAs have lower coding potentials, fewer exon numbers, lower expression levels and shorter transcript lengths (**Supplementary Figure 1**). We screened out 2,752 DE mRNAs and 1,515 DE lncRNAs (**Supplementary Table 4**), accounting for 21.05% and 7.92% of total identified mRNAs and lncRNAs, respectively. More specifically, three pairwise comparisons between the stages (13D *vs*. 1D, 26D *vs*. 1D, 26D *vs*. 13D) were performed to identify differentially expressed mRNAs and lncRNAs, and obtained 2354, 1891, 15 DE mRNAs (**Figure 2A**) and 878, 1084, 11 DE lncRNAs (**Figure 2B**), respectively. Both differentially expressed mRNAs and lncRNAs numbers between 1D and elder age stage (13D or 26D) are much greater than those between 13D and 26D. Furthermore, three DE genes (AKR1D1, VSIG1 and BCL2A1) overlapped in all three comparisons (**Figure 2C**).

**Figure 3:**
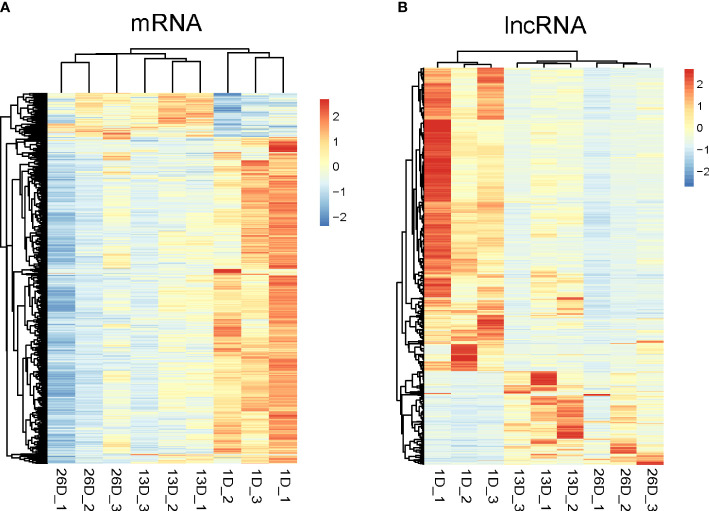
Hierarchical clustering analysis of differentially expressed mRNAs and lncRNAs in pigeon BFs. Heatmap representing the Z-score for normalized expression values of DE mRNAs **(A)** and lncRNAs **(B)**. The heatmap is drawn with Pretty Heatmap at ImageGP.

Subsequently, hierarchical clustering analyses were conducted on the expression profiles of differentially expressed mRNAs and lncRNAs. As depicted in **Figure 3A–B**, DE mRNAs and lncRNAs expression data in pigeon BFs clustered into two groups according to developmental stages, respectively. The elder squabs (13D and 26D) were clustered into a subgroup, and separated from 1-day-old pigeon squabs. It implied that the developmental time leads to the different mRNA and lncRNA transcriptomes in pigeon BFs.

### Expression patterns and potential functions of DE mRNAs during BF development

**Figure 4:**
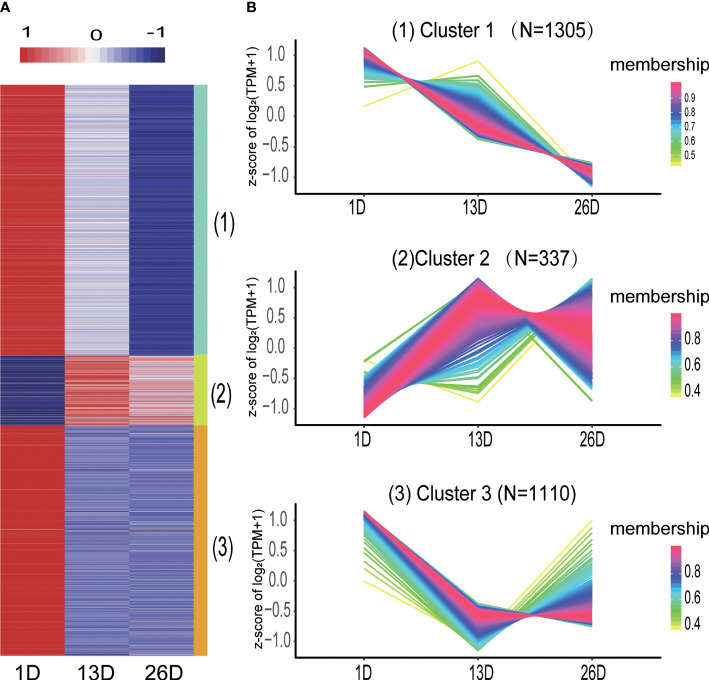
Distinct expression patterns of differentially expressed mRNAs across the BF development stages. **(A)**. The heatmap shows the expression patterns of the three clusters. Each row represents a mRNA. The color indicates the Z-score of normalized expression values. **(B)**. Cluster analysis of the DE mRNA expression patterns across the BF development stages. Membership values indicate the goodness of fit for mRNAs in a particular cluster.

The expression patterns of DE mRNAs in different BF developmental stages were analyzed by using the TCseq R package. DE mRNAs were divided into three categories (**Figure 4A–B**, **Supplementary Table 5**). Cluster 1 comprised 1305 mRNAs and their expression levels in 26D are lower than those in 1D and 13D. Cluster 2 containing 337 mRNAs showed higher expression in 13D or 26D compared to 1D, while the expression of 1110 mRNAs in cluster 3 was rapidly downward from 1D to 13D and then increased or slowly decreased in 26D (**Figure 4B**).

**Figure 5:**
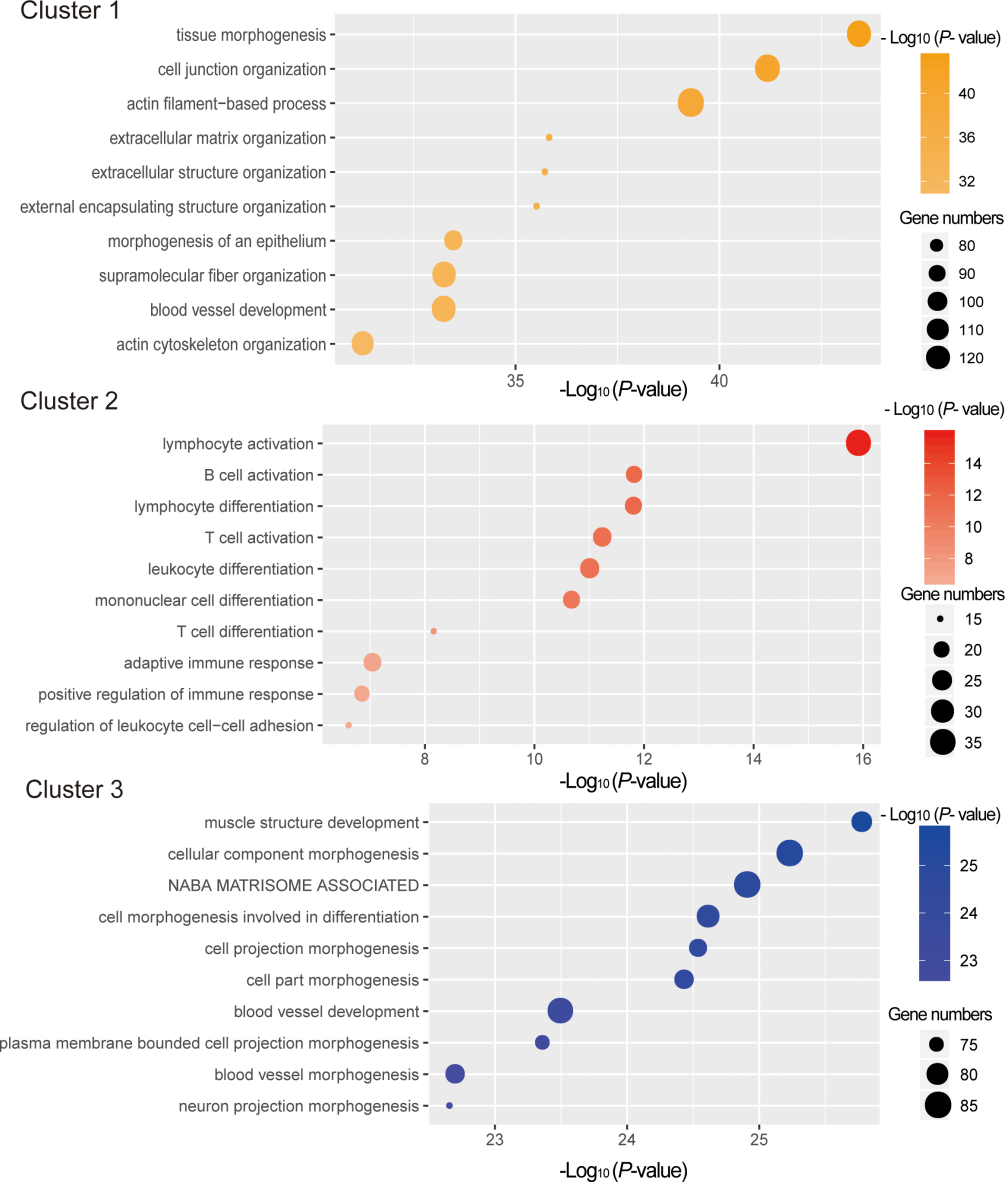
Function enrichment analysis of DE mRNAs in different clusters. The x-axis indicates –log (P - value). The y-axis indicates functional categories.

**Figure 6:**
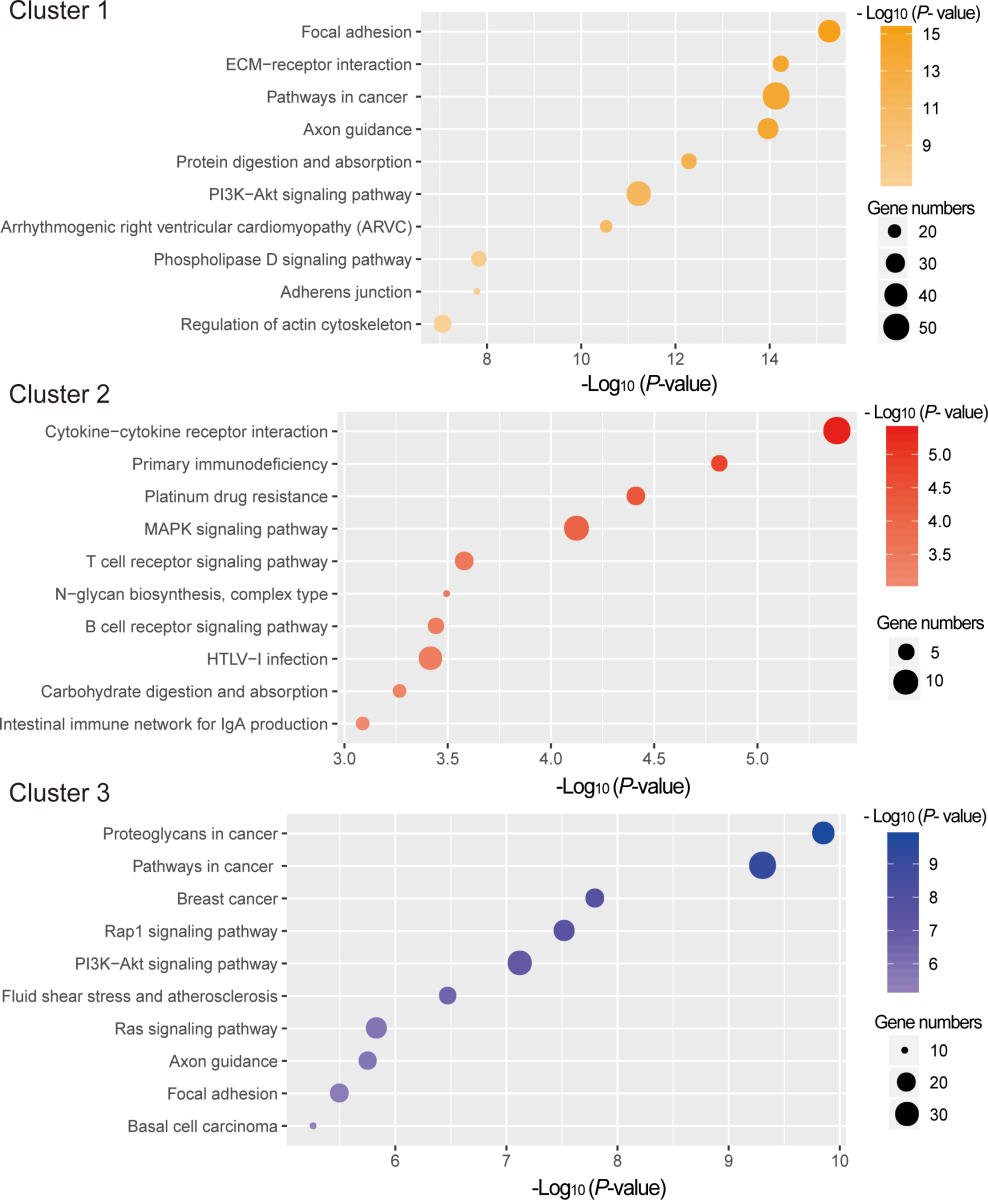
KEGG pathway analysis for DE mRNAs in different clusters. The x-axis indicates –log (P - value). The y-axis indicates KEGG pathways.

To explore the potential functions of the mRNAs in different clusters, functional enrichment analyses were conducted with Metascape. As depicted in **Figure 5**, genes in cluster 1 and cluster 3 were mainly enriched for the GO terms related to tissue morphogenesis and development, whereas those in cluster 2 were enriched in the immune related GO terms, such as lymphocyte activation (GO:0046649), B cell activation (GO:0042113), lymphocyte differentiation (GO:0030098), leukocyte differentiation (GO:0002521), etc (**Supplementary Table 6**). KEGG pathway enrichment analysis also showed that enriched pathways for genes in cluster 2 are also linked with immune, including Cytokine-cytokine receptor interaction (ko04060), Primary immunodeficiency (ko05340), T cell receptor signaling pathway(ko04660), B cell receptor signaling pathway(ko04662), etc (**Figure 6**, **Supplementary Table 6**).

### PPI network and MCODE enrichment analysis

**Figure 7:**
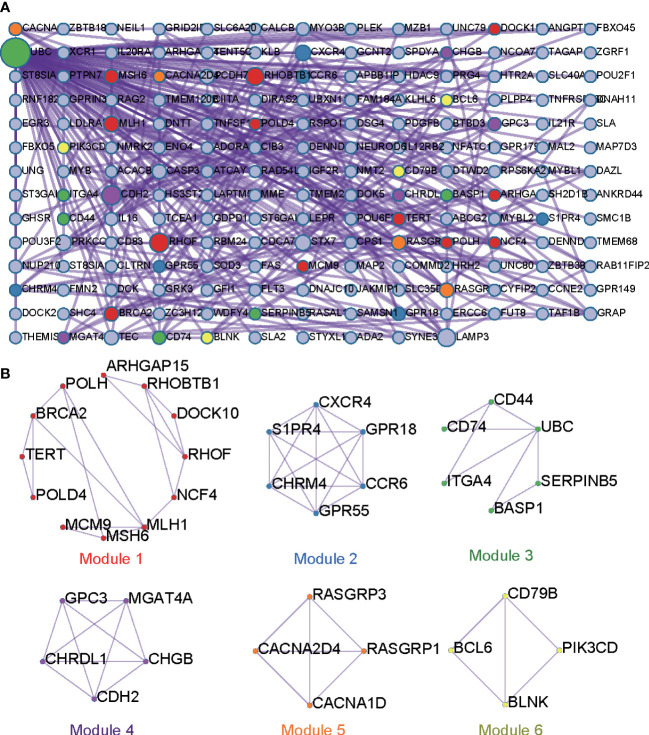
PPI interaction networks and core modules for DE genes in cluster 2. **(A)**. PPI interaction network. **(B)**. Modules of PPI.

Given genes in cluster 2 are closely related to immune, these genes were employed to construct PPI network. (**Figure 7A**). The PPI network of cluster 2 consisting of 179 nodes and 315 edges was constructed in the different protein interaction databases including STRING (18), BioGrid (19), OmniPath (20), InWeb_IM (21). MCODE algorithm was employed to identify highly connected network components from the PPI network. Six significant modules containing 37 genes were screened from our constructed PPI network by MCODE (**Figure 7B**, **Supplementary Table 7**). These significant vital modules showed functions including B cell activation, lymphocyte differentiation, positive regulation of leukocyte cell-cell adhesion, DNA repair, ion homeostasis, etc (**Supplementary Table 7**).

### Expression patterns and potential functions of DE lncRNAs during BF development

**Figure 8:**
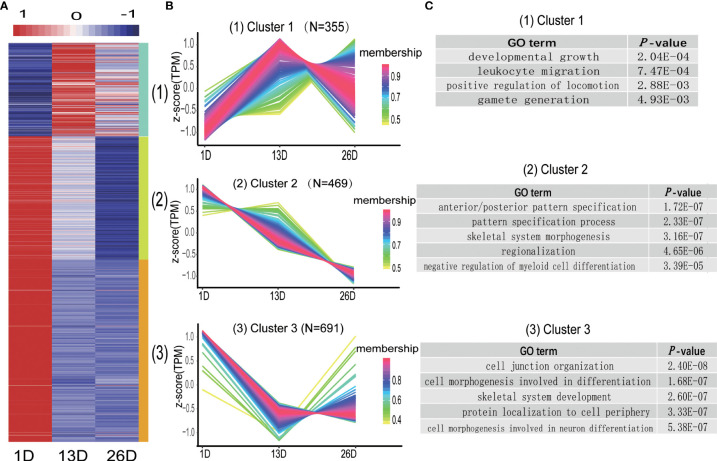
Distinct expression patterns of differentially expressed lncRNAs across the BF development stages. **(A)**. The heatmap shows the expression patterns of the 3 clusters. Each row represents a lncRNA. The color indicates the Z-score of normalized expression values. **(B)**. Cluster analysis of the DE lncRNA expression patterns across the BF development stages. Membership values indicate the goodness of fit for lncRNAs in a particular cluster. **(C)**. Representative enrichment categories of target genes of DE lncRNA in different clusters.

Like DE mRNAs, the expression patterns of DE lncRNAs were also divided into three categories (**Figure 8A, B**, **Supplementary Table 5**). Cluster 1 comprising 355 lncRNAs showed higher expression level in 13D or 26D compared to 1D. Cluster 2 contains 469 lncRNAs, and their expression levels in 26D are lower than those in 1D and 13D. Expression of 691 lncRNAs in cluster 3 was rapidly downward from 1D to 13D and then increased or slowly decreased in 26D (**Figure 8B**). To explore the potential functions of the lncRNAs in different clusters, cis target genes of DE lncRNAs in each cluster were predicted and subjected to functional analysis. As a result, 21, 72 and 129 potential target genes were detected in cluster 1-3, respectively (**Supplementary Table 8**). Target genes of DE lncRNA in cluster 1 are involved in developmental growth (GO:0048589), leukocyte migration (GO:0050900) and positive regulation of locomotion (GO:0040017), etc. Those in cluster 2 and 3 are mainly enriched for GO terms associated with development and morphogenesis (**Figure 8C** and **Supplementary Table 9**).

### qPCR validation

**Figure 9:**
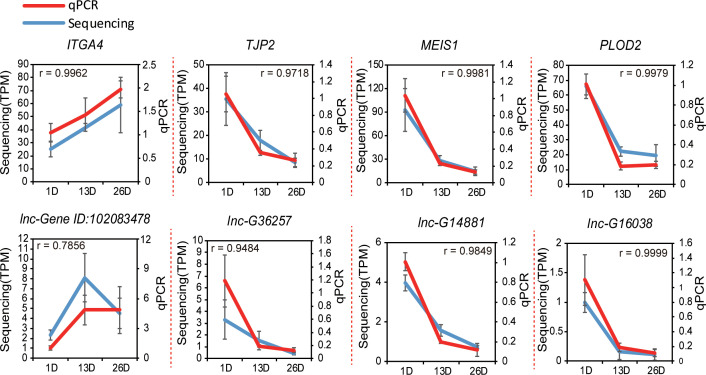
Validation of the sequencing data using qPCR. Four randomly selected protein coding genes and four lncRNAs in pigeon BFs were validated by real-time qPCR (n = 3). The Pearson product–moment correlation coefficient (r) was calculated using R.

To confirm the RNA-seq results, we randomly selected eight DE genes and lncRNAs to conduct qPCR assay using three independent samples. The results were in line with our sequencing result (Pearson r =0.960 ± 0.073) (**Figure 9**), which highlighted the reliability of our sequencing data.

## Discussion

BF is acknowledged as a central humoral immune organ unique to poultries. BF development goes through three phases, including the rapid growth phase (first 3 weeks after hatch), the plateau phase, and the regression phase (22). Three development stages (1d, 13d, and 26d) were selected in this study, representing newly hatched, rapid development and plateau stage, respectively. We identified 13,072 mRNAs and 19,129 lncRNAs from three development phases of squab BFs. BF development process is accompanied by changes in gene expression. Specifically, 2,752 mRNAs and 1,515 lncRNAs were identified as differentially expressed. Moreover, it was found that DE mRNAs and lncRNAs numbers between 1D and elder age are much greater than those between 13D and 26D. It might be associated with greater BF size or weight difference between 1D and 13D or 26D.

Generally, the gene expression pattern is well connected with gene function (23). In our study, gene expression pattern analysis found that identified DE protein coding genes could be clustered into three groups. GO functional enrichment analysis found genes in cluster 1 and cluster 3 were implicated in the tissue morphogenesis and development. Of special interest and importance is DE genes in another cluster (cluster 2) which have higher expression level on 13D or 26D compared to 1D. Their functional annotations are principally involved in immune system process-related GO categories, including lymphocyte activation, B cell activation, lymphocyte differentiation, leukocyte differentiation and T cell differentiation, etc. It has been well established that BF is the location of B lymphocyte differentiation and maturation in avians. BF provides an environment to support the differentiation of B lineage cells (24), consistent with our functional enrichment analysis results. Furthermore, KEGG pathway enrichment analysis revealed that these DE genes in cluster2 are involved in Cytokine-cytokine receptor interaction, B cell receptor signaling pathway and MAPK signaling pathway, which have been demonstrated to be relevant to BF development and functions (2, 25). Representative DE genes involved in immune system process contain CD79b, BCL6, CD83, CD74, etc. Among these, CD79b also known as B29 and Ig beta, is an essential component of the B cell receptor. CD79b exhibited strict B cell-specific expression (26) and expressed from early in B-cell development to the plasma cell stage (27). CD79b plays a critical role in B cell development. Targeted knockout of CD79b gene severely disrupts pre-B-cell development (28, 29). During chicken BF development, CD79b gene is also identified as differentially expressed and shows a similar expression tendency with our results (2). Bcl-6 is not only involved in the regulation of effector and memory differentiation of B lymphocytes, but also influences effector and memory differentiation in CD4+ T cells and CD8+ T cells (30). CD83 participates in the regulation of T- and B-lymphocyte maturation and in the regulation of their peripheral responses (31). CD74 also exerts modulation functions in the process of T-cell and B-cell developments (32). These critical genes in BF exhibit higher expression levels at 13D and 26D compared with 1D, implying BF with larger size might play more positive roles in lymphocyte differentiation and development. Several lines of evidence also support this view (11, 33).

lncRNAs are crucial regulators of cell differentiation and development (34), which participate in the regulation of gene expression at multiple levels. By interacting with RNA, chromatin, and protein, lncRNAs modulate mRNA stability, chromatin structure, and the function of proteins (35). lncRNAs have the potential to regulate immune cell function and development. For instance, Tiago F. Brazão and colleagues identified 4516 lncRNAs expressed in 11 stages of mice B-cell development and activation (36). Our results found that in addition to some protein-coding genes related immune system process and tissue morphogenesis, 1,515 lncRNAs are identified as differentially expressed during BF development. DE lncRNAs show similar three expression patterns with differentially expressed protein-coding genes. Of note, cis target genes of DE lncRNAs in cluster 1 were enriched in leukocyte migration, positive regulation of locomotion, development growth, etc. These enriched GO catergories are closely linked with functional process of B lymphocytes. It is well documented that fully differentiated B lymphocytes would migrate from the BF to peripheral lymphoid organs to colonize, reproduce and perform important immune functions (5). Representative target genes of DE lncRNAs include IL16, CXCR4 and ITGA4, etc. IL-16, also known as lymphocytic chemoattractant factor (LCF), can regulate the migration and proliferation of normal leukocytes (37). The chemokine receptor CXCR4 is abundant on naive lymphocytes. It is a critical regulator of cell migration (38). ITGA4 is a member of the integrin family of transmembrane receptors, which is involved in generating signals pivotal for cell migration (39). Combining our results with the functions of these target genes, it can be speculated that lncRNAs might participate in the regulation of pigeon BF development and B lymphocyte function.

Based on our findings that immune-related mRNAs and lncRNAs were identified as differentially expressed, we speculated that, like other poultries, pigeon BF plays important roles in B lymphocyte development and differentiation. The reasons why pigeon squabs subjected to bursectomy didn’t exhibit a decline of humoral immune responsiveness were possibly related to the following facts: Firstly, unlike other poultries, parent pigeons have the capability to secrete crop milk, which contains immunoglobulin, protein and fat, etc (40, 41). By means of feeding crop milk, pigeon parents could transfer immunoglobulin to squabs (42). Secondly, emigration of B cells from bursa to periphery lymphoid tissues may start around the time of hatch (43). Thirdly, Approximately 5% of peripheral B cells in the newly hatched bird appear to be derived from extrabursal precursors (44).

Overall, in this study we presented the results of lncRNA and mRNA expression profiling in pigeon BF across three different development stages and highlighted their potential biological function. Our findings will provide insight into the transcriptional difference involving in pigeon BF development and enhance the understanding the physiological functions of squab BF.

## Materials and methods

### Preparation of experimental animals and tissues

White King pigeon squabs were purchased from the Feng Mao pigeon breeding plant (Mianyang, China). Thirty-nine pigeon squabs were grouped into three stages (13 replicates for each period, the age of 1 day (1d), 13 days (13d), 26 days(26d). After measuring the body weight, the pigeon squabs were anesthetized with diethyl ether and euthanatized. Then, the BF of each pigeon was isolated and weighed. BF index was calculated using the formula: BF index = BF weight/body weight ×100. And the samples for subsequent lncRNA and mRNA sequencing were frozen in liquid nitrogen and stored at -80°C refrigerator.

### Histomorphological examination of BF

BFs were fixed in 4% paraformaldehyde for over 24h and dehydrated in graded ethanol. Subsequently, BFs were embedded in paraffin and subjected to a microtome (Leica, Wetzlar,Germany). Serial 5μm-thick slices were prepared and stained with H&E, and taken photos with a digital camera.

### RNA extraction and high-throughput sequencing

Total RNA was respectively isolated from frozen BF samples collected from nine female pigeon squabs across the three developmental stages (three biological replicates each) by HiPure Universal RNA Kit (Magen, Guangzhou, China) according to the manufacturer’s protocol. The integrity of total RNA was evaluated using an Agilent 2100 Bioanalyzer. The RNA concentrations were assessed with a Nanodrop 2000 spectrophotometer (Thermo Scientific, Massachusetts, USA). Each total RNA sample was first treated to remove ribosomal RNA using the Ribo-Zero™ kit (Epicentre,Madison, WI, USA). The rRNA-depleted RNA was used to prepare the stranded-specific RNA-seq library following the manufacturer’s standard procedures. Subsequently, prepared cDNA libraries were sequenced by an Illumina NovaSeq 6000 platform and the paired-end reads were obtained. All the RNA-seq data have been deposited in NCBI database with accession number GSE183791.

### Assembly of pigeon transcripts and identification of lncRNA

High-quality clean reads were obtained by removing reads containing adapters and low-quality reads from the raw data. These clean reads were mapped to the pigeon genome (Cliv_1.0 from NCBI) using STAR (45) (v.2.6.0c). Mapped reads were assembled to transcripts using the Cufflinks (46) (v.2.1.1) software. The transcripts (≤250 bp or FPKM < 0.1) were first excluded. The remaining transcripts were merged and compared to the reference genome by the meta-assembly tool TACO software (v0.7.3). Those transcripts unannotated by protein-coding genes (PCGs) were retained as putatively lncRNA transcripts. Subsequently, Coding Potential Calculator (CPC2) and PfamScan (47) were applied to predict the coding potential of these transcripts. After removing transcripts with coding capacities, the remaining transcripts were identified to be long non-coding RNAs.

### Differential expression and cluster analysis

Quantification of mRNA and lncRNA expression in each sample was calculated in TPM (transformed transcripts per kilobase million) scale by Kallisto (48) (version 0.43.0). Next, mRNAs with TPM > 0.5 and lncRNAs with TPM > 0.1 in at least one library were considered expressed. Differential expression analysis between two stages was performed using edgeR (49)(Release 3.13) in the OmicShare tools (www.omicshare.com/tools), with |log2(fold change) |>1 and FDR <0.05. The expression patterns of differentially expressed mRNAs and lncRNAs were characterized by R package TCseq, respectively (50).

### Target gene prediction of DE lncRNA

To explore the possible function of DE lncRNAs, cis target genes of lncRNAs were predicted. DE genes located 100 kb upstream and downstream of lncRNAs were extracted. Pearson’s correlation coefficients (PCC) between DE mRNAs and DE lncRNAs were calculated with the Hmisc package in R. The lncRNA-mRNA pairs with |PCC| > 0.9 and P < 0.05 were predicted as co-regulated.

### Functional enrichment analysis

The DE mRNAs and cis target genes of DE lncRNAs were subjected to functional enrichment analyses by using Metascape (http://metascape.org/gp/index.html) online tool with the default parameters set (51). The GO terms and KEGG pathways with P < 0.05 were considered significantly enriched.

### Protein-protein interaction network analysis of DE genes

Protein-protein interaction (PPI) networks were constructed by using the Metascape tool with default parameters. Next, MCODE algorithm was employed to identify highly connected network components from the PPI network. Functional enrichment analysis was conducted for each MCODE component, and the three best-scoring terms (by P-value) were retained as the functional description of the corresponding modules.

### Quantitative real-time PCR validation

The total RNA was reverse transcribed respectively by PrimeScript™ RT reagent Kit with gDNA Eraser (Takara, Otsu, Japan) following the manufacturer’s recommendation. The qRT-PCR assays were performed on a CFX96 Real-Time PCR Detection System (Bio-Rad, Hercules, CA) using SYBR Premix Ex Taq II (Novoprotein, Shanghai, China) by the manufacturer’s protocols. Relative mRNA and lncRNA levels were calculated by the 2^-△△Ct^ method. The primer sequences are in **Supplementary Table 1**.

### Statistical analysis

BF weight and index were analyzed and compared for statistically significant difierences using one-way analysis of variance (ANOVA) in R, respectively. The significance level for the statistical analysis was P < 0.05.

## Data availability statement

The datasets presented in this study can be found in online repositories. The names of the repository/repositories and accession number(s) can be found below: NCBI Gene Expression Omnibus (GEO) repository under GEO accession GSE183791.

## Ethics statement

This study was reviewed and approved by the Institutional Animal Care and Use Committee in the College of Animal Science and Technology, Sichuan Agricultural University, Sichuan, China.

## Author contributions

Conceptualization: XW and ML. Methodology: LJ, JM, LZ, YL and QT. Validation: HG and AJ. Investigation: JW, QP and FY. Writing-original draft preparation: XW and JW. Writing-review and editing: XL and HT. Visualization: SH. Funding acquisition: ML. All authors contributed to manuscript revision, read, and approved the submitted version.

## Funding

This work was supported by the Sichuan Science and Technology Program (2021YFYZ0009), the Ya’an Science and Technology Program (21SXHZ0022).

## Acknowledgments

We thank the High-Performance Computing Platform of Sichuan Agricultural University and Ya’an Big Data Industrial Park for providing computing resources and support that have contributed to these research results.

## Conflict of interest

The authors declare that the research was conducted in the absence of any commercial or financial relationships that could be construed as a potential conflict of interest.

## Publisher’s note

All claims expressed in this article are solely those of the authors and do not necessarily represent those of their affiliated organizations, or those of the publisher, the editors and the reviewers. Any product that may be evaluated in this article, or claim that may be made by its manufacturer, is not guaranteed or endorsed by the publisher.
