# Supplementary material for: Transcriptome analysis revealed the roles of long non-coding RNA and mRNA in the bursa of Fabricius during pigeon (Columba livia) development
Source: Front Immunol. 2022 Jul 25;13:916086. doi: 10.3389/fimmu.2022.916086 (PMC9357926; doi:10.3389/fimmu.2022.916086)
Supplement: Supplementary file 1 [file DataSheet_1.zip › Supplementary Figures and Tables.docx]

Supplementary Material

# Supplementary Figures and Tables

## Supplementary Figures


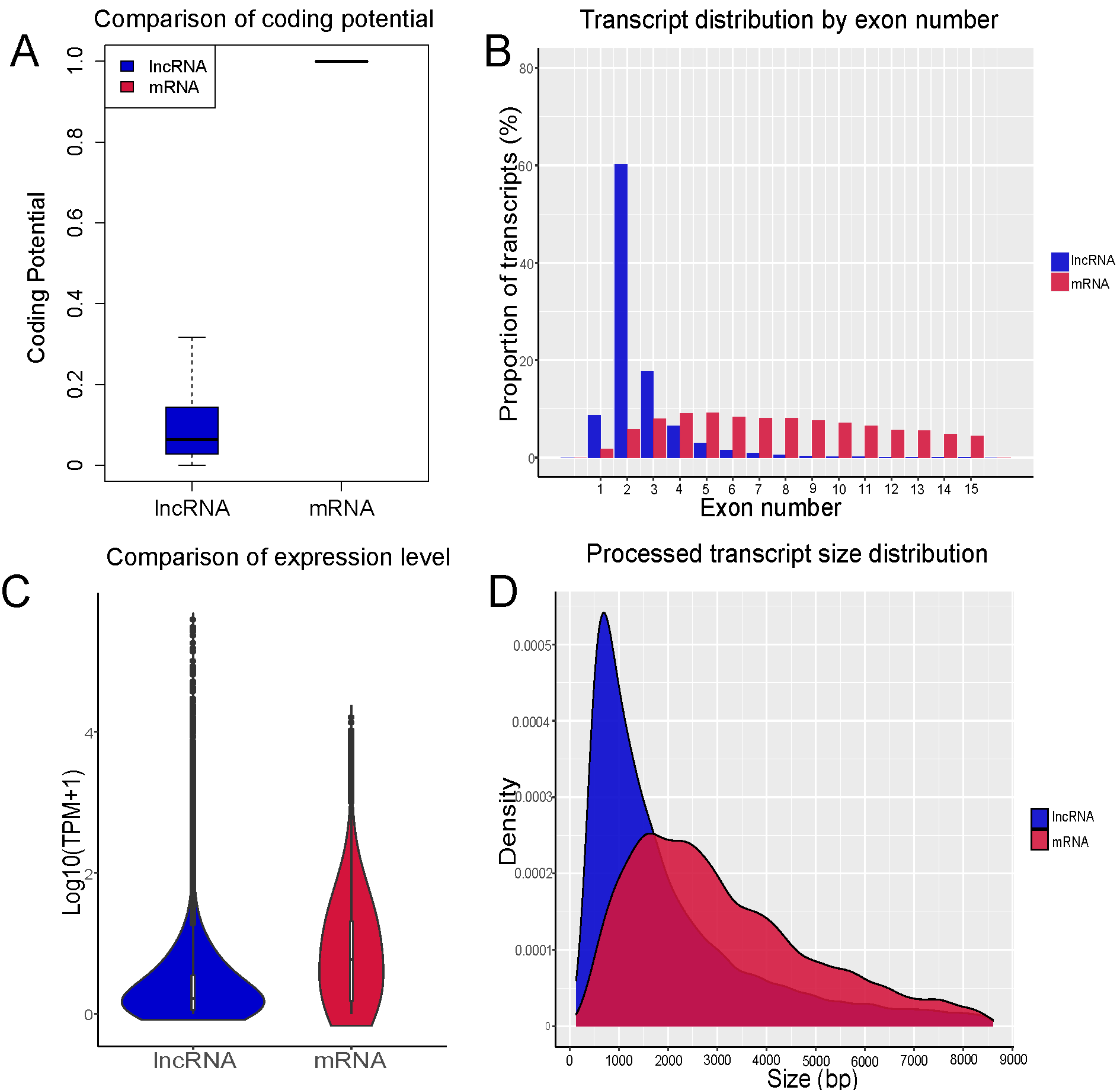


**Supplementary Figure 1.** Transcriptional characterization of lncRNAs and mRNAs in pigeon BF LncRNA and mRNA expression changes during BF development.

## Supplementary Tables

**Supplementary Table 1.** Primer sequences of the qPCR assays

| **Name** | **Primer sequence (5' - 3')** | **Product length(bp)** |
| --- | --- | --- |
| *ITGA4* | F:GAGAAAACCACGGGTCATGC | 80 |
|  | R:TGATCCAGGGGCTCCCATTA |  |
| *TJP2* | F:TGTCATTCCTCACAAGGCCC | 112 |
|  | R:TCTGCCACCAGAAACTGCAA |  |
| *MEIS1* | F:ATCTACGGACACCCCCTGTT | 113 |
|  | R:TCAGAGGAGCAAACATCGCC |  |
| *PLOD2* | F:GAAAGGTGGTGAGCTGCCTA | 50 |
|  | R:GTCGAACTTTCTGCCCTCCA |  |
| *lnc-Gene ID:102083478* | F:AAAAGCACACGAGCACCAC | 50 |
|  | R:AGCGTTCAGTTCGATTGGCT |  |
| *lnc-G36257* | F:GTCACAGCATCTGCGTTTGG | 50 |
|  | R:CTCAAGAGTTCCACCACGCT |  |
| *lnc-G14881* | F:TAGCGGGGTCCAGACACTTA | 76 |
|  | R:GGTTCTCCATCCAAGGGCAA |  |
| *lnc-G16038* | F:AGGCTCGGGAAAACCCATTT | 84 |
|  | R:AAGCCAAGACTGCGAACTGA |  |
| *β-actin* | F:GTGGATCAGCAAGCAGGAGT | 101 |
|  | R:TCATCACAAGGGTGTGGGTG |  |

Abbreviations: F, forward; R, reverse.

**Supplementary Table 2.** Summary of data information

| **Sample Name** | **Raw Data(Gb)** | **Clean Data (Gb)** | **Proportiaon of Q30 (%)** | **Raw Reads** | **Clean Reads** | **Mapped Reads** | **Map Ratio (%)** |
| --- | --- | --- | --- | --- | --- | --- | --- |
| 1D_1 | 11.33 | 11.16 | 94.46 | 37,778,932 | 37,197,457 | 34,703,113 | 93.29 |
| 1D_2 | 11.15 | 10.93 | 93.03 | 37,180,382 | 36,434,384 | 29,698,257 | 81.51 |
| 1D_3 | 11.96 | 11.84 | 93.38 | 39,877,427 | 39,468,076 | 34,859,653 | 88.32 |
| 13D_1 | 10.48 | 10.28 | 94.21 | 34,940,163 | 34,274,961 | 32,070,896 | 93.57 |
| 13D_2 | 10.86 | 10.71 | 94.89 | 36,190,725 | 35,699,893 | 33,564,299 | 94.02 |
| 13D_3 | 10.28 | 10.22 | 92.83 | 34,280,233 | 34,070,831 | 31,467,408 | 92.36 |
| 26D_1 | 12.06 | 11.93 | 94.2 | 40,197,841 | 39,759,791 | 35,570,330 | 89.46 |
| 26D_2 | 13.62 | 13.5 | 94.16 | 45,412,013 | 45,009,336 | 41,888,723 | 93.07 |
| 26D_3 | 11.82 | 11.7 | 93.78 | 39,390,592 | 38,995,717 | 34,665,530 | 88.90 |
